# Theoretical model of passive mode-locking in terahertz quantum cascade lasers with distributed saturable absorbers

**DOI:** 10.1515/nanoph-2023-0657

**Published:** 2024-02-01

**Authors:** Lukas Seitner, Johannes Popp, Michael Haider, Sukhdeep S. Dhillon, Miriam S. Vitiello, Christian Jirauschek

**Affiliations:** TUM School of Computation, Information and Technology, Technical University of Munich (TUM), D-85748 Garching, Germany; Laboratoire de Physique de l’Ecole Normale Supérieure, ENS, Université PSL, CNRS, Sorbonne Université, Université de Paris, Paris, France; NEST, CNR – Istituto Nanoscienze and Scuola Normale Superiore, Piazza San Silvestro 12, 56127, Pisa, Italy; TUM Center for Quantum Engineering (ZQE), Technical University of Munich (TUM), D-85748 Garching, Germany

**Keywords:** terahertz, quantum cascade laser, graphene, frequency comb, passive mode-locking, Maxwell–Bloch

## Abstract

In research and engineering, short laser pulses are fundamental for metrology and communication. The generation of pulses by passive mode-locking is especially desirable due to the compact setup dimensions, without the need for active modulation requiring dedicated external circuitry. However, well-established models do not cover regular self-pulsing in gain media that recover faster than the cavity round trip time. For quantum cascade lasers (QCLs), this marked a significant limitation in their operation, as they exhibit picosecond gain dynamics associated with intersubband transitions. We present a model that gives detailed insights into the pulse dynamics of the first passively mode-locked QCL that was recently demonstrated. The presence of an incoherent saturable absorber, exemplarily realized by multilayer graphene distributed along the cavity, drives the laser into a pulsed state by exhibiting a similarly fast recovery time as the gain medium. This previously unstudied state of laser operation reveals a remarkable response of the gain medium on unevenly distributed intracavity intensity. We show that in presence of strong spatial hole burning in the laser gain medium, the pulse stabilizes itself by suppressing counter-propagating light and getting shortened again at the cavity facets. Finally, we study the robustness of passive mode-locking with respect to the saturable absorber properties and identify strategies for generating even shorter pulses. The obtained results may also have implications for other nanostructured mode-locked laser sources, for example, based on quantum dots.

## 1 Introduction

The operation of lasers in a mode-locked state is highly desirable in many applications, such as metrology and sensing [1], [2]. In the spectral domain, mode-locking is associated with the formation of comb spectra consisting of equidistant discrete lines. The realization of laser frequency combs was awarded the Nobel Prize in physics, indicating its importance in present and future applications [2], [3]. Fundamental research of light–matter interactions can be performed for many different timescales, down to femtoseconds, using ultrashort laser pulses [4]. Specifically, in the terahertz (THz) and mid-infrared (mid-IR) portion of the electromagnetic spectrum, the dynamics of gas molecules may be explored as they exhibit roto-vibrational modes at these frequencies. The corresponding optical transitions can be used for molecular fingerprint detection in sensing and spectroscopy [5]. While spectroscopic platforms have been realized in this fingerprint region (e.g., [6], [7]), the absence of compact and inexpensive short pulse emitters hinders the implementation of short pulse applications [8]. Recently, a passively mode-locked THz quantum cascade laser (QCL) was demonstrated in a compact configuration, which may fill this gap [9]. Here, we present a model that gives a detailed understanding of the mode-locking dynamics in this device, as is required for an improved understanding of the underlying mechanisms and a targeted design and optimization of such devices. The obtained results may also have implications for the design of other passively mode-locked semiconductor lasers with short gain recovery times, for example, based on quantum dots (QDs).

In general, amplitude-modulated mode-locking can be achieved by active injection of an optical or electrical signal, or passively by a suitable modification of the laser cavity [1]. Passive mode-locking does not require dedicated circuitry and allows for more compact setups, thus having advantages for practical applications. Furthermore, the minimum achievable pulse duration is inherently shorter than that achievable via active mode-locking [1]. In general, passive mode-locking can be realized through saturable absorbers [10], [11] or nonlinear phase shifts [12], [13]. Today’s standard theory of passive mode-locking explains the dynamics of pulse formation very well when the gain recovers more slowly than the cavity round trip time [1]. However, the model does not cover self-starting passive mode-locking when the gain dynamics become too fast. QCLs extend the spectral regime covered by interband semiconductor lasers to mid-infrared and THz frequencies and exhibit gain recovery times significantly faster than the cavity round trip time [14], [15]. Thus, passive mode-locking of QCLs was assumed to be rather difficult, and research focused mainly on frequency-modulated (FM) combs in Fabry–Pérot cavities and soliton generation in ring cavities, e.g., [16–20]. The possibility of comb generation in the low THz region by difference frequency generation of mid-infrared radiation inside a single QCL has been explored in experiment and simulation [21]–[23]. But also direct generation of THz frequency combs can be achieved by dispersion engineering, even though the resulting waveform typically features both frequency and amplitude modulation rather than single isolated pulses [24]. Several theoretical works have been dealing with the possibility of amplitude-modulated mode-locking in fast gain media, based on self-induced transparency [25], a combination of active and absorbing regions [26], [27], coherent and memory effects [27]–[29], or optical injection [30]. However, the generation of passively mode-locked pulses has been experimentally achieved only very recently [9]. In this device, multilayer graphene (MLG) stripes embedded alongside the top contact serve as a distributed saturable absorber for the THz radiation and drive the laser in the pulsed state. It has been proven experimentally that THz saturable absorption in graphene depends on the number of layers (*n*_layer_) and that transparency modulation increases with *n*_layer_ [31], [32]. Hence, MLG is employed to enhance the saturable absorption effect, which is known to be the key mechanism for passive mode-locking. Apart from that, there is also a technical limitation in the use of single-layer graphene: its clean adhesion onto the top surface stripes of a processed QCL is worse than in the case of MLG, which conversely ensures good adhesion, flatness, and preserves a clean process. In a previous report [9], we presented numerical results based on a Maxwell-density matrix approach, considering nine quantized levels per period in the active region. This model showed very good agreement with experimental data but, due to its complexity, did not provide an intuitive understanding of the mode-locking dynamics [9]. Starting from this framework, we develop a simplified description, which still contains the relevant dynamics but offers an intuitive understanding of the pulse formation and stabilization. This allows us to pin down the necessary properties of absorber and gain for pulse formation. Since the Maxwell–Bloch equations are widely used for describing nano-optoelectronic devices [33], our model provides a basis for investigating related effects in other types of semiconductor lasers.

## 2 Theoretical model

One major conclusion drawn in the seminal paper by Haus is that the “model of a fast saturable absorber and a fast laser medium does not give self-starting mode-locking solutions” [34]. Notably, for intermediate situations with gain recovery times on a comparable timescale as the round trip time, self-starting mode-locking is not excluded by the Haus theory, but this case is less amenable to analytical solutions and has less been studied in literature (see, e.g., [1], [28], [29]). However, for gain recovery times much shorter than the round trip time the conditions under which self-starting passive mode-locking can still be obtained become increasingly restricted (see, e.g., [26]). In the limiting case of fully recovered gain and loss after a round trip, the existence of a net gain window exclusively at the position of the pulse is not compatible with a self-starting scenario, requiring that the unsaturated gain exceeds the unsaturated loss. Thus, this section focuses on additional effects due to counter-propagation and associated spatial hole burning, not included in the Haus approach but relevant especially in semiconductor lasers, which can lead to additional pulse stabilization.

First, we introduce the optical propagation equation, which is coupled to the absorber model. Then, we describe the active region dynamics based on the Bloch equations with spatial hole burning (SHB) and extract an analytical formula for the direction-dependent gain.

**Figure 1:**
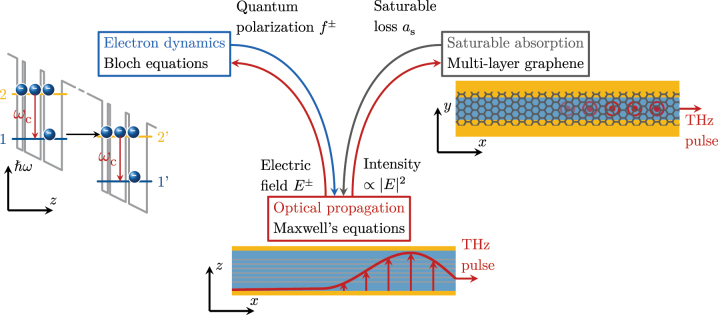
Visualization of the semi-classical, dynamical model, which consists of three domains and their interactions. The electron dynamics of a fast gain medium are described by the Bloch equations. A possible realization is the quantum-well heterostructure of a QCL active region. The reduced two-level model accounts for coherent optical transitions and incoherent scattering between adjacent periods. The polarization and electric field terms introduce coupling between the active region description and the optical propagation equation, which is derived from Maxwell’s equations. A saturable absorber model accounting for the distributed multilayer graphene completes the system description. It interacts with the optical field via an intensity-dependent loss.

The overall model implemented in the present work is summarized in Figure 1.

### 2.1 Optical propagation in presence of saturable absorption

The time evolution of the electric field is governed by a propagation equation, which is derived from Maxwell’s equations as [1], [35], [36]

(1) $$v_{\text{g}}^{-1}\partial_{t}E^{\pm }=\mp \partial_{x}E^{\pm }+f^{\pm }-lE^{\pm }+\left(D_{f}-i\frac{\beta_{2}}{2}\right)\partial_{t}^{2}E^{\pm }.$$

The electric field is in the slowly varying envelope approximation (SVEA) described as the sum of two counter-propagating components $E\left(x,t\right)=\sum_{\pm }\frac{1}{2}\left[E^{\pm }\exp\left(\pm i\beta_{0}x-i\omega_{\text{c}}t\right)+\text{ c.c.}\right]$ , where c.c. denotes the complex conjugate, *β*_0_ is the propagation constant, *ω*_c_ represents the center frequency, and *v*_g_ denotes the group velocity. The term *D*_*f*_ introduces the effect of gain bandwidth, while *β*_2_ models the background material’s group velocity dispersion. The field components of Eq. (1) interact with the saturable absorber via the loss term *lE* ^±^, and with the gain medium via the intersubband polarization *f* ^±^. These two driving forces of the system need to be balanced in order to achieve the pulsed state. Notably, this propagation equation holds significant similarity to the master equation derived by Haus [1], regarding the treatment of saturable loss, dispersion, and gain bandwidth *D*_*f*_. However, it does not explicitly contain a nonlinearity term [1], as, e.g., considered for the modeling of QCL FM combs [36]–[38], such that all nonlinear effects in the here-discussed system arise from the standard Bloch equations with SHB [39] and the addition of the saturable absorber.

Due to the outstandingly high electron mobility and ballistic transport properties in cryogenic graphene [40], [41], we assume absence of SHB in the saturable absorber. Furthermore, because of the peculiar band structure [42] and complex optoelectronic dynamics [43], [44], we choose a compact phenomenological saturable absorber model for the graphene at THz frequencies [9], [44]–[46]. For finite recovery times, saturable absorption is typically described by [35]:

(2) $$\partial_{t}a_{\text{s}}=-\frac{I}{\tau_{\text{a}}I_{\text{s,a}}}a_{\text{s}}-\frac{a_{\text{s}}-a_{1}}{\tau_{\text{a}}}.$$

The saturable loss then enters the propagation equation via the relation

(3) $$l\left(x,t\right)=\frac{1}{2}\left[a_{0}+a_{\text{s}}\left(x,t\right)\right].$$

As can be seen, the overall loss is split into a constant waveguide and material contribution *a*_0_, and a saturable part *a*_s_. Due to the distribution of graphene over the whole cavity, this part is a function of space and time *a*_s_(*x*, *t*) and has a maximum value of *a*_1_. Therefore, Eq. (2) accounts for loss saturation with increasing intensity $I\left(x,t\right)$ and recovery with a characteristic time *τ*_a_. The remaining parameter in this model is the effective saturation intensity *I*_s,a_, defined as the value of the intracavity intensity that saturates the graphene loss to *a*_s_ = *a*_1_/2.

### 2.2 Modeling of electron dynamics

To describe the QCL gain medium, we aim for a model that is simple enough to give intuition but still accounts for the physical mechanisms that enable passive mode-locking. From a very detailed multilevel density matrix approach [33], [47], as used in [9], we construct a more compact two-level Bloch model [33], [39], [48], [49]. The corresponding parameters are determined by preserving the crucial properties of the resulting gain, i.e., maximum, recovery time, and bandwidth. Thus, no parameter fitting is needed. The state of the two-level system is determined by the coherence *η*, inversion *w*, and inversion grating *w* ^+^ [39]. For bi-directional optical propagation, as present in Fabry–Pérot cavities, this inversion grating needs to be considered, as it accounts for optical interference effects like SHB. This effect is known to play a major role in multimode and comb operation of QCLs and other nanostructured lasers and is, therefore, explicitly considered in our model [16], [36], [38], [39], [50], [51]. In homogeneously broadened laser systems, the decay of quantum coherence between the laser levels is typically the fastest process in the system. In a THz QCL, it is usually about 10 times faster than the gain recovery time. By assuming it to be infinitely fast, i.e., performing the adiabatic elimination of *η* [52], the Bloch equation system can be simplified, allowing for a more intuitive understanding. After restructuring the equations and disregarding minor contributions, they read as:

(4) $$\eta_{21}^{\pm }=-\frac{i}{2\hbar \gamma_{2}}d_{21}\left[wE^{\pm }+w^{\pm }E^{\mp }\right],$$

(5) $$\partial_{t}w=-\frac{d_{21}^{2}}{\hbar^{2}\gamma_{2}}w\left(|E^{+}{|}^{2}+|E^{-}{|}^{2}\right)-\gamma_{1}\left(w-w_{\text{eq}}\right),$$

(6) $$\partial_{t}w^{+}=-\frac{d_{21}^{2}}{\hbar^{2}\gamma_{2}}wE^{+}{\left(E^{-}\right)}^{*}-\gamma_{1}^{\prime }w^{+}.$$

Here $w^{-}={\left(w^{+}\right)}^{*}$ , *d*_21_ is the optical dipole moment, *γ*_1_ denotes the gain recovery rate, *γ*_2_ is the dephasing rate, and *w*_eq_ represents the equilibrium inversion. Further, $\gamma_{1}^{\prime }$ is the recovery time of the inversion grating, which is affected by charge carrier diffusion,

(7) $$\gamma_{1}^{\prime }=\gamma_{1}+4\beta_{0}^{2}D,$$

were *D* denotes the diffusion constant. A more detailed derivation of these equations is given in the Supplementary Information Section I.B. The resulting polarization of this system, which enters the electric field propagation equation, is then given by

(8) $$f^{\pm }=i\alpha \eta_{21}^{\pm },$$

with

(9) $$\alpha =\frac{\Gamma n_{3D}\omega_{\text{c}}d_{21}}{c_{0}\varepsilon_{0}n_{0}}.$$

Here, Γ denotes the overlap factor, *n*_3D_ is the space averaged doping density, and *c*_0_, *ɛ*_0_, and *n*_0_ are the vacuum speed of light, permittivity, and material refractive index, respectively. We notice from Eqs. (4)–(9) that in presence of the inversion grating *w* ^+^, the gain is not only saturated by an intensity $\propto \left(|E^{+}{|}^{2}+|E^{-}{|}^{2}\right)$ but also an interference term $E^{+}{\left(E^{-}\right)}^{*}$ is present. In fact, when setting *w* ^+^ = 0 in, then Eq. (4), Eqs. (4) and (5) correspond to an implementation of the gain into the Haus model in analogy to Eq. (2) (compare to Supplementary Information Section I.D). Therefore, our model with SHB can be seen as a direct extension to the Haus theory.

As a consequence of the adiabatic elimination, gain curvature and dispersion get neglected. However, since a finite gain bandwidth is necessary for stable mode-locked solutions, it is reintroduced via *D*_*f*_ in the propagation equation (1) [1], [35]. The derivation of *D*_*f*_ is outlined in the following subsection, along with further analytical considerations, that are crucial to describe and understand the pulse dynamics. Furthermore, by adjusting *v*_*g*_ and *β*_2_ in Eq. (1), gain dispersion may be considered, but this effect turns out to be of minor importance in our case.

### 2.3 Direction-dependent gain model

Our approach to calculate the gain is based on taking the field propagation equation, Eq. (1), and equating the intersubband polarization to an optical amplification, which yields the forward and backward gain coefficient

(10) $$g^{\pm }=\pm 2R\left(\frac{f^{\pm }}{E^{\pm }}\right).$$

This direction-dependent gain means that left and right traveling fields might experience different amplification strengths. This approach may be counterintuitive at first sight, but in the field of nonlinear optics, counter-propagating effects can significantly influence the system dynamics [53]. For the implementation of the gain bandwidth in Eq. (1), we use the relation

(11) $$D_{f}=\frac{g_{\text{s}}}{2\gamma_{2}^{2}}$$

with a direction-averaged gain value

(12) $$g_{\text{s}}=\frac{g^{+}I^{+}+g^{-}I^{-}}{I^{+}+I^{-}},$$

where $I^{\pm }=\frac{1}{2}\varepsilon_{0}c_{0}n_{0}{\left|E^{\pm }\right|}^{2}$ are the intensities of each field component.

Recent theoretical works have investigated the role of such counter-propagating interactions for different operating regimes of QCLs [20], [38], [36]. In the case of monochromatic excitation (*∂*_*t*_ *w* = 0), we can derive an analytic expression for the direction-dependent gain from the adiabatic Bloch equations, Eqs. (4)–(6) (see Supplementary Information Section I.C):

(13) $$g^{\pm }=\alpha \frac{d_{21}}{\hbar \gamma_{2}}\left(1-\frac{2I^{\mp }}{2I_{\text{s,g}}\gamma_{1}^{\prime }/\gamma_{1}+I^{+}+I^{-}}\right)w.$$

We observe that for two fields *E* ^±^ with different amplitudes, the stronger field component experiences a higher gain than the weaker counter-propagating field. This shows that the field with higher amplitude couples more efficiently with the gain medium. In a standard linear cavity, this effect is usually not essential, as facet reflections and irregular intensity fluctuations due to SHB dominate the dynamics. By adding the distributed saturable absorber, uneven intensity distributions arise, and the effect becomes more pronounced. For comparison, in a ring cavity, one field component completely decays in the absence of balancing mechanisms [19], [20], which is a direct result of the directive gain.

To get a better understanding of this phenomenon, we study two steady-state scenarios analytically, starting from expression (13). At first, we consider the case where the intensities in both field directions are equal $\left(I^{-}=I^{+}\right)$ , which happens at the facet of the cavity if we assume full reflectivity. This condition yields the maximum optical interference between the counter-propagating fields and effectively reduces the saturation intensity by the factor (see Supplementary Information Section I.C)

(14) $${\left.I_{\text{s,g}}\right|}_{I^{-}=I^{+}}=\frac{2\gamma_{1}^{\prime }}{2\gamma_{1}^{\prime }+\gamma_{1}}I_{\text{s,g}}.$$

Assuming small diffusion and thus close-to-maximum SHB in the gain medium, we obtain $\gamma_{1}^{\prime }\approx \gamma_{1}$ , and consequently *I*_s,g_ is effectively reduced to 2/3 of its original value. For a suitable choice of parameters, the value of the gain can drop below the losses, even though exceeding it in an unsaturated state. In the case of a short pulse hitting the facet, the finite reaction times of gain and loss take effect, and a net loss window opens up at the trailing edge, leading to pulse shortening and suppression of subsequent fluctuations.

In the second case, we assume that one intensity direction is significantly stronger than the other one, *I* ^∓^ ≪ *I* ^±^. This resembles the case where a high-intensity pulse travels along the cavity, and the counter-propagating intensity mainly consists of noise. Obviously, in this situation, no significant optical interference is present, and thus *I*_s,g_ remains at its initial value. However, since in this case (see Supplementary Information Section I.C)

(15) $${\left.g^{\mp }\right|}_{I^{\mp }\ll I^{\pm }}=\left(1-\frac{2I^{\pm }}{2I_{\text{s,g}}\gamma_{1}^{\prime }/\gamma_{1}+I^{\pm }}\right)g^{\pm },$$

the gain gets reduced for the counter-propagating weak background. For example, if the pulse intensity is as strong as the saturation intensity, *I* ^±^ = *I*_s,g_, and we again assume negligible diffusion $\left(\gamma_{1}^{\prime }\approx \gamma_{1}\right)$ , then the resulting gain for the counter-traveling field would drop to *g* ^∓^ = *g* ^±^/3. In steady-state, the overall gain is clamped to the loss, meaning that the fainter field experiences a net loss.

For THz QCLs, $\gamma_{1}^{\prime }\approx \gamma_{1}$ is typically a valid approximation, since the comparably low frequency results in a small *β*_0_ in Eq. (7), and the charge carrier diffusion is negligible at cryogenic temperatures. In semiconductor lasers that operate at higher frequencies and temperatures, like mid-IR QCLs, interband cascade (IC), or QD lasers, the relative impact of diffusion is increased. Nevertheless, SHB is still known to influence the dynamics of these lasers [39], [50], [51], [54], [55]. Thus, both previously described concepts are valid for these structures, and the impact of saturation intensity reduction and directional noise suppression can be quantitatively evaluated by inserting suitable parameters into Eqs. (14) and (15). Furthermore, the presented framework can be applied to saturable absorbers where a certain amount of SHB is present, to evaluate if the here-described pulse stabilizing mechanisms are still effective. This can be achieved by applying Eqs. (4)–(6), or respectively Eq. (13), also to the absorber, and evaluating the direction-dependent net gain $g^{\pm }-\left(a_{0}+a_{\text{s}}^{\pm }\right)$ . For modeling passive mode-locking in QD lasers [56], the inclusion of SHB would require adapted Maxwell–Bloch equations [51], since the decay of the population grating in QD media is not well captured by the conventional diffusion model.

In the following, to reveal the full dynamics of the system, the coupled equations of gain, absorber, and intensity are studied numerically.

## 3 Numerical results and discussion

For our dynamic simulations of a mode-locked THz QCL, realistic parameter values extracted from experiment [9], literature [32], [44], [45], [57], and Monte-Carlo simulations [58] are used in the model. An overview of these values is given in Tables 1 and 2. Here, we describe the gain in terms of macroscopic parameters (maximum gain *g*_0_, saturation intensity *I*_s,g_, recovery time 1/*γ*_1_, coherence time 1/*γ*_2_). The microscopic contributions leading to these values (optical dipole moment, scattering rates, etc.) can be found in the Supplementary Information Section II. The losses (*a*_0_, *a*_1_) have been evaluated by dedicated electromagnetic simulations [9], and experiments show that the absorber recovers within *τ*_a_ = 2 ps to 3 ps [9], [32], [57], so we choose an intermediate value. Although the nonlinear optical properties of graphene have been successfully exploited to affect THz QCL operation [9], [59], [60], assessing its saturation intensity at THz frequencies appears to be complex, and different values can be found [32], [44]–[46]. Furthermore, the polarization direction of the electric field compared to the graphene surface has an influence on the saturation dynamics [44]. However, a polarization rotation possibly happening at the metal/graphene interface is not included in our one-dimensional model. Thus, we treat *I*_s,a_ as the only fitting parameter in our model. Notably, the recovery times of gain and absorber are almost equal in the case considered here, and the gain maximum is slightly above the sum of background and saturable loss. A discussion on the robustness of the device against parameter variations will be given below and in the Supplementary Information.

**Table 1:** Parameters of gain.

1/*γ*_1_	1/*γ*_2_	*g*_0_	*I*_s,g_
2.63 ps	0.167 ps	22.5 cm^−1^	13 × 10^7^ W m^−2^

**Table 2:** Parameters of absorber.

*τ*_a_	*a*_0_	*a*_1_	*I*_s,a_
2.5 ps	11.8 cm^−1^	10.5 cm^−1^	5 × 10^7^ W m^−2^

### 3.1 Simulation of experimental setup

**Figure 2:**
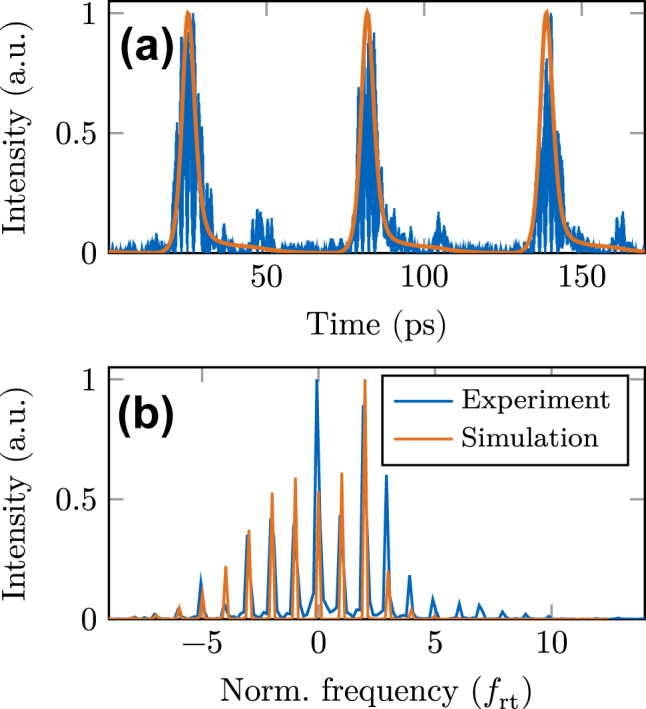
Comparison of simulation results to experiment. (a) Time trace of the normalized intensity, outcoupled from the mode-locked laser. (b) Power spectrum of the electric field, normalized to the round-trip frequency with an offset of roughly 3 THz.

At first, we directly compare simulation results with time-resolved measurements, as given in [9]. In addition to the above stated parameters, we use realistic values for the mirror reflectivity (*R* = 0.7 [61]) and the diffusion constant in the gain medium (*D* = 20 cm^2^ s^−1^). The resulting time trace of the outcoupled intensity is given in Figure 2(a) and the corresponding spectra of the fields in Figure 2(b). A clear, background-free pulse operation with roughly 6 ps full-width-half-max (FWHM) duration is visible in the experiment and simulation, which is linked to a frequency comb in the spectral domain. This very good agreement supports the validity of the adiabatic simulation approach and also the reduction of the original nine-level system, which is self-consistently extracted from charge-carrier transport simulations [9], to an equivalent two-level model. The measured and simulated frequency combs, given in Figure 2(b), exhibit around 10 lines in a 10 dB power range, with the experimental one having slightly more contributions at the higher frequency side. This small deviation may be due to the effect of after-pulsing in the measured time trace of Figure 2(a), which is not prominent in the simulations. When the integrated graphene absorber is removed (compare Supplementary Information Section III.A), the laser output becomes completely unlocked in both experiment and simulation. This straightforwardly leads to the conclusion that the graphene saturable absorber drives the QCL into the pulsed state.

**Figure 3:**
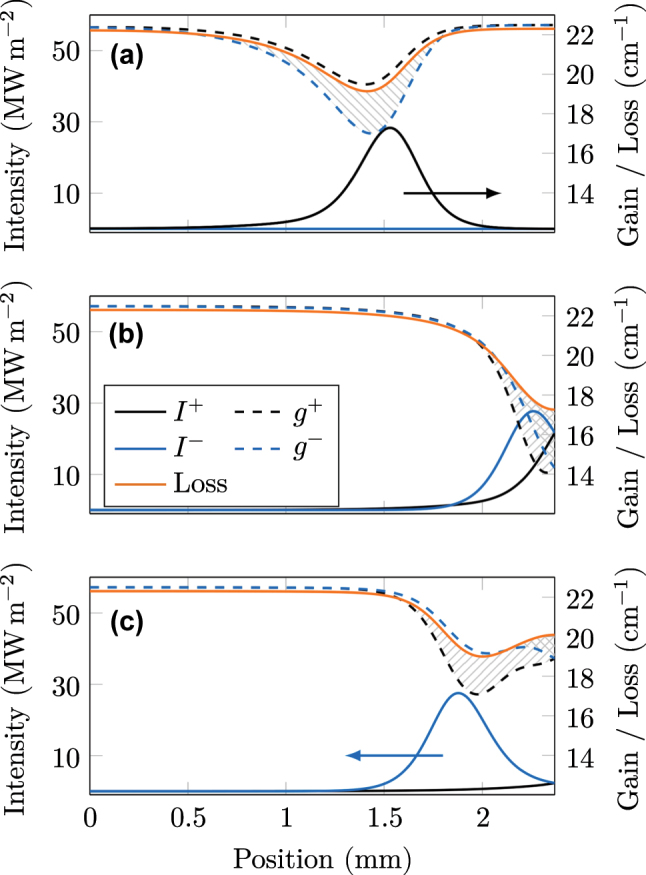
Steady-state intra-cavity pulse dynamics. Shaded areas refer to loss regions for a certain direction (striped) or both directions (checkered). (a) When the pulse is located in the middle of the cavity, gain and loss are slightly saturated and almost equal. The weak counter-propagating field experiences a significant loss. (b) At the facet of the cavity, right and left traveling intensities are equally strong. This leads to a net loss window for both directions. (c) When the pulse has left the facet, the strong gain saturation due to optical interference is still present. As the absorber is independent of this effect, a significant loss window is created.

In order to get the best visualization of the dynamics that keep a generated pulse stable (compare Section 2.3), we set the facet reflectivity to unity, while converting the outcoupling to a distributed loss [49], and neglect charge carrier diffusion in the quantum-well heterostructure. The resulting steady-state behavior of the intracavity intensity, gain, and loss is shown in Figure 3. In Figure 3(a), a pulse is located in the middle of the cavity, filling a significant part and traveling toward the right facet. The presence of this intensity peak leads to a dip in the distributed gain and loss. As both have fast recovery dynamics with nearly equal time constants, they have returned to their initial values at the cavity facets. The right-traveling intensity (*I* ^+^) forms the pulse and grows along its path, as the right-traveling gain (*g* ^+^) slightly exceeds the loss everywhere. The intensity of the left-traveling wave (*I* ^−^) is by orders of magnitude smaller and gets further dampened as the left-traveling gain (*g* ^−^) is smaller than the loss. This scenario corresponds to Eq. (15) of the analytical model. When the pulse hits the facet, as in Figure 3(b), both gain components saturate to lower values than the loss, effectively creating an overall net loss window. This net loss at the facet is crucial for pulse stability and highly similar to saturable absorbing mirrors used in common passive mode-locking setups [1], [10]. At the interface, right and left traveling electric field components are present in equal strength, leading to strong optical interference. As discussed along Eq. (14), for this scenario, the saturation behavior of the gain is strongly affected by the inversion grating term *w* ^+^. In contrast, the absorber in our model is only influenced by the intensity and thus saturates less. When the pulse has changed its direction, now traveling to the left, as illustrated in Figure 3(c), its tail is still prone to the overall net loss window and thus gets cut off. In this way, the pulse can sustain its shape, even in the presence of group velocity dispersion. Although this mechanism prevents the pulse from broadening, there may still be a background formation due to the finite net gain in the rest of the cavity.

This way, undesired background fluctuations may form in the middle and at the left side of the cavity, while the pulse is reshaped at the right facet. This weak field will counter-propagate toward the pulse and, at some point, reach the same location of the cavity. In this situation, the strong pulse absorbs the weak fluctuations because they experience a net loss, as described above. We emphasize that this is an intrinsic effect of the quantum system that arises when taking into account the population grating arising from optical interference in the Bloch equations. However, adding a saturable absorber with suitable properties is the crucial ingredient to generate the conditions for pulsed operation, where this effect is revealed. The described dynamics of one complete round trip are animated in Supplementary Movie 1.

Another manifestation of directional gain is symmetry-breaking in ring lasers [19], [20], [62]. There, through spontaneous emission during the formation process, one field direction randomly grows stronger, thus reducing the available gain for the other direction. With increasingly asymmetric intensities, this effect gets more pronounced, resulting in a complete decay of the counter-propagating field. Consequently, this results in unidirectional operation unless a balancing mechanism, such as backscattering, is present [20], [63].

**Figure 4:**
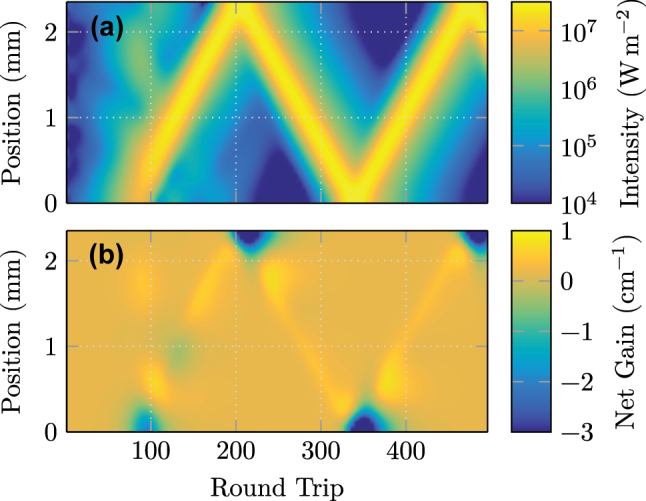
Visualization of (a) intracavity intensity and (b) net gain during the formation process. The zigzag trace arises from a slight intentional undersampling of the round trip frequency, such that the pulse is visualized at each point of the cavity.

This tendency to unidirectional operation is similar to the formation dynamics in the here-discussed case of passively mode-locked pulses, where a kind of symmetry breaking is also present. In Figure 4(a), the transient behavior of the intracavity power is depicted for the first 500 round trips over the whole cavity, starting from random noise. During the first 50 round trips, the weak initial fluctuations get slowly amplified and, due to *D*_*f*_, filtered into a smooth but unevenly distributed intensity. Then, between ≈50 and 100 round trips, a nonlinear process takes place, rapidly amplifying randomly generated power accumulations by orders of magnitude, while hardly affecting the remaining intensity. Notably, this is only possible due to the presence of a saturable absorber, which is fast enough to follow the gain dynamics, and at the same time saturates slightly more easily. In this specific simulation, a clear pulse forms at cavity positions close to 0 mm. However, a significant amount of distributed, unlocked intensity is still present in the cavity. The dynamics of background absorption and symmetry breaking are visible between round trip 100 and 200. After the background has collided several times with the pulse (twice per round trip), it has lost intensity by several orders of magnitude. In contrast to that, the pulse gains energy throughout this period of time. Therefore, these formation dynamics can be seen as a type of symmetry breaking. After the buildup process, i.e., for times >250 round trips, the pulse completely dominates the dynamics of the laser.

In Figure 4(b), the net direction-averaged gain *g*_n_ = *g*_s_ − (*a*_s_ + *a*_0_) inside the cavity is depicted. We observe similar behavior as described alongside Figure 3. In the middle of the cavity, there is a small positive net gain present, and its maximum follows the intensity peak. The loss for the counter-propagating field is not visible, as its impact on the direction-averaged gain value *g*_s_ in Eq. (12) scales with its weak intensity. However, when the pulse reaches one of the facets, a significant loss window emerges, as both intensity directions significantly contribute to the saturation. As outlined above, this effect acts as a pulse shortening and stabilizing mechanism. The pulse formation dynamics sampled at each round trip are visualized in Supplementary Movie 2.

The effect of spontaneous emission noise on the laser dynamics has been tested by adding small perturbations of the electric field at every grid point, implemented similarly as in [64]. Considering the detailed active region model with nine levels, spontaneous emission causes fluctuations of the pulse peak power with a standard deviation of ≈1 %. The observed beatnote linewidth is well below the numerical frequency resolution of ≈2 MHz. As the cavity round trip frequency is at ≈20 GHz, the timing jitter of the pulses should thus be at least 10,000 times smaller than the round trip time.

### 3.2 Investigation of operating regimes

After discussing the mode-locking dynamics in detail for a certain set of parameters, we study the robustness of the system against deviations in the absorber properties. The saturation intensities of gain and absorber (*I*_s,g_ and *I*_s,a_) indicate the values at which the respective variable clamps to half of its maximum. Therefore, the absorber gets saturated by a lower intracavity intensity than the gain, which is an important relation, as discussed below. Further, in the parameter sets of Tables 1 and 2, we see that the gain maximum *g*_0_ is just slightly above the sum of the overall losses *a*_0_ + *a*_1_. If the difference between unsaturated gain and loss increases, the generation of passively mode-locked pulses breaks down, as background formation cannot be suppressed anymore. However, evaluating the mode-locking regime in terms of current, which is commonly used as the adjustable variable in measurements, loosens this tight condition. Assuming that in steady state, electronic transitions exactly compensate for the number of lost photons, we can calculate a photon-driven current density *J*_ph_ in terms of the losses and intensity

(16) $$J_{\text{ph}}=\left\langle \left(a_{0}+a_{\text{s}}\left(x,t\right)\right)I\left(x,t\right)\right\rangle \frac{e_{0}L_{\text{p}}}{\hbar \omega_{\text{c}}}.$$

**Figure 5:**
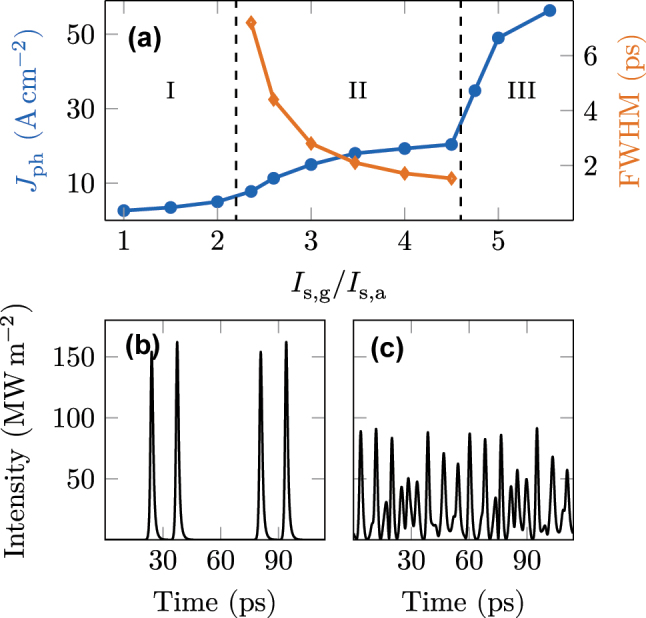
(a) Dependence of photon-driven current on the variation of the absorber saturation intensity. Three different regimes appear. I Single mode and weak amplitude modulation. II Single pulsed state. Decreasing the absorber intensity increases the current. Decreasing full-width-half-max (FWHM) pulse width indicates higher intensity pulses. III When the absorber bleaches too easily, unlocked multipulsing and chaotic states appear. (b) Double pulsing at the border between region II and III. (c) Chaotic emission of region III.

Here, the overall intensity $I\left(x,t\right)$ is given as the sum of the left- and right-traveling components $I\left(x,t\right)=I^{+}\left(x,t\right)+I^{-}\left(x,t\right)$ . Further, the operator ⟨⋅⟩ denotes the spatial and temporal average over one round trip, and *L*_p_ is the length of one QCL period. By this evaluation, we observe that a current density range of *J*_ph_ ≈ 15 A cm^−2^ yields single pulsed states, and multipulsed states extend the narrow beatnote range by about another ≈10 A cm^−2^. Therefore, even with the restriction that the unsaturated gain must not exceed the unsaturated loss too far, a significant bias-current range opens up, where the mode-locked operation may be observed experimentally. Regarding realistic QCLs, in addition to this photon-driven contribution, dark charge carrier transport holds a significant portion of the overall current. This contribution may further extend the current range where a narrow beatnote is detected, as it has only a minor influence on the field dynamics. As a comparison, the experimental device showed a threshold current density of ≈200 A cm^−2^, and a narrow beatnote regime was detected up to ≈75 A cm^−2^ above this threshold. A more detailed analysis of the photon-driven current and different operational states of the simulations is given in Figure 5. Since the absorber saturation intensity *I*_s,a_ depends on the exact overlap of the transverse optical field mode with the graphene stripes in the cavity, *I*_s,a_ is nontrivial to determine for our one-dimensional simulation approach. Therefore, we evaluate the robustness of the system against deviations of *I*_s,a_ while keeping the gain parameters unchanged. We obtain three different possible states that the laser may assume, and we can calculate the photon-driven current in these regimes, which is also directly proportional to the output power. In regime I, where both quantities have comparable values, the gain quickly clamps to the losses in the whole cavity, only allowing for single-mode operation or weak amplitude modulations. As this state exhibits weak power, the photon-driven current is on the order of 5 A cm^−2^, and the output power some hundreds of microwatts. When the absorber bleaches ≈2.2 times more easily than the gain, the laser switches to region II, the single pulsed state. Up to a factor of 4.5 difference between *I*_s,g_ and *I*_s,a_ single pulse mode-locking is possible, showing that the system is tolerant against deviations of this parameter. The associated photon-driven current covers a range between ≈10 and 25 A cm^−2^. A decreasing value of the pulse’s FWHM is observed when reducing *I*_s,a_, which goes along with higher peak intensities. Further, the pulse duration is also affected by the presence and strength of chromatic dispersion [65]–[68]. For the results shown in Figure 5(a), a value of *β*_2_ = 5 × 10^−23^ s^2^ m^−1^ was used as parameter in Eq. (1). Ideal dispersion compensation would thus lead to shorter pulses, as already mentioned in [9], while stronger dispersion broadens the pulse (compare Supplementary Information Section III.B).

Regarding the variation of saturation intensities, the case in which the absorber bleaches ≈5 times more easily than the gain results in unlocked multipulsing or chaotic behavior (region III), as depicted in Figure 5(c). However, the first data point of this region in Figure 5(a), at ≈35 A cm^−2^, shows a double-pulse, see Figure 5(b), with a clear beatnote still present.

### 3.3 Transition to standard theory

**Figure 6:**
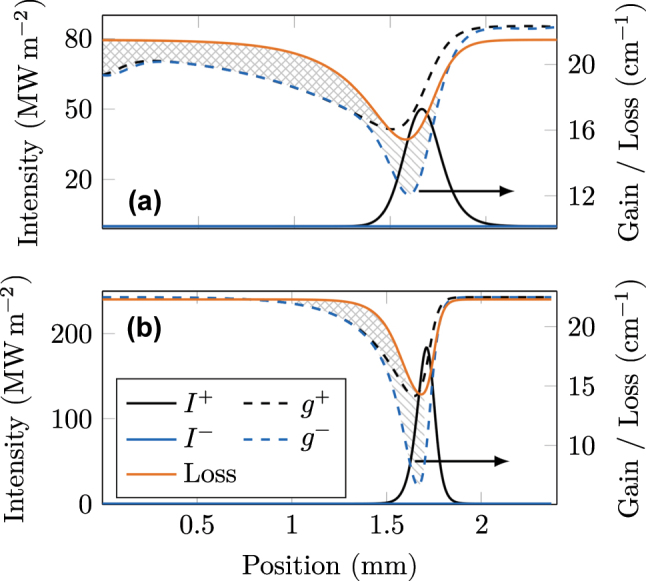
Converged pulse when the absorber recovers significantly faster than the gain. (a) The gain recovers within 10.5 ps, the absorber within 3 ps. (b) The gain recovers within 2.6 ps, the absorber within 1 ps. In both cases, a net loss window is always present behind the pulse.

We have now discussed in detail the case where the recovery times of gain and absorber are very similar, *τ*_a_ ≈ *τ*_g_, as this comes closest to the experiment [9]. If the recovery time of the absorber exceeds the one of the gain (*τ*_a_ ∼ 10 % larger than *τ*_g_), the rapid nonlinear amplification, visible in Figure 4(a) between ≈50 and 100 round trips, cannot take place and, therefore, pulsed operation is not supported. We note that the ratio of recovery times may change due to different possible realizations of gain media (THz, mid-IR QCLs, IC, or QD lasers) and possible improvements in the recovery speed of saturable absorbers. Furthermore, different values of gain recovery times in THz QCLs have been reported in literature, ranging from 2 ps to several tens of ps [15], [69]–[71]. In the Supplementary Infromation, we depict the maximum ratio of gain and loss over the ratio of gain and absorber recovery times that still yields stable pulses. In order to extend the coverage of our model for the cases with longer gain recovery time, we show results of pulsed operation for *τ*_g_ = 10.5 ps and *τ*_a_ = 3 ps in Figure 6(a). The remaining used parameters, as well as a color map of the spatiotemporal intensity, can be found in the Supplementary Information Section III.C. Notably, in this situation, there is a large loss window behind the pulse, allowing for very strong background absorption. Thus, a region with significant gain in front of the pulse can be balanced without yielding chaotic behavior. In fact, this specific simulation was carried out using *a*_0_ = 11.0 cm^−1^, such that the gain maximum exceeds the unsaturated loss by *g*_0_ − (*a*_0_ + *a*_1_) = 1 cm^−1^. Therefore, assuming this rather long gain recovery time, the model reacts less sensitively to the gain/loss ratio than for similar recovery times, as described above. This observation goes along with the discussion at the very beginning of Section 2, as this simulation rather approaches the scenario of intermediate gain recovery times. Hence, for *τ*_g_ = 10.5 ps, the strong demands on the gain and absorber parameters may be significantly relaxed, while pulses can still be obtained.

Additionally, there is the possibility left where the absorber is significantly faster than the gain, *τ*_a_ < *τ*_g_, while the gain recovery time remains at *τ*_g_ = 2.6 ps. Therefore, we set *τ*_a_ = 1 ps and leave the remaining parameters as in Tables 1 and 2. In Figure 6(b), the resulting steady-state distributions of intensity and gain are depicted. A single pulse emerges that is significantly shorter, with higher peak intensity, as compared to the initial simulation (Figure 3). In Figure 6(b), a net loss window emerges behind the pulse. Therefore, the tail of the pulse gets constantly cut off, while the leading edge sees a large amount of gain. Thus, photons are created at the front of the pulse and get re-absorbed at its end. As a result of this asymmetry, the pulse velocity is higher than the intrinsic group velocity of the medium, which determines the speed of background fluctuations. The dominating dynamics of this case are highly similar to the one of laser self-pulsations, described in [72]. The superluminal motion of the pulse additionally enables the loss window to suppress the amplification of background fluctuations throughout the whole cavity and, thus, to sustain pulsed operation.

## 4 Summary and conclusion

To summarize, we have presented a model of single pulse passive mode-locking in lasers with fast recovery time, i.e., faster than the cavity round trip time, such as THz QCLs. The presence of an incoherent distributed saturable absorber, realized by graphene stripes embedded along the top contact, can lead to pulse formation if it saturates with a suitable intensity and recovers at least equally fast as the gain. The incoherent optical properties of graphene and its high charge carrier mobility, suppressing SHB, make it an ideal choice for the absorber and enable the formation of a net loss window at the cavity facets. An optical interference term appearing in the gain description, associated with SHB, enables the suppression of a continuous wave background. The model shows a certain robustness against parameter variations and allows mode-locking in a significant current density bias range. Furthermore, the case when the absorber is faster than the gain is investigated. A shorter pulse emerges, which is followed by a pronounced loss window. Thus, the stabilizing effect is not restricted to the cavity interfaces, and interference effects become less important. Our work increases the understanding of experimentally realized passive pulse formation in lasers with fast gain dynamics, such as THz QCLs, and provides a guide on how shorter pulses can be achieved. The described model may also be relevant for the design of other mode-locked lasers with quantum-engineered active regions, such as IC and QD lasers.

## Supplementary Material

Supplementary Material Details
