# Supplementary material for: Theoretical model of passive mode-locking in terahertz quantum cascade lasers with distributed saturable absorbers
Source: Nanophotonics. 2024 Feb 1;13(10):1823–34. doi: 10.1515/nanoph-2023-0657 (PMC11501480; doi:10.1515/nanoph-2023-0657)
Supplement: Supplementary file 1 — Supplementary Material Details [file j_nanoph-2023-0657_suppl_001.pdf]

Supplementary Material – Theoretical model of passive mode-locking in terahertz quantum cascade lasers with distributed saturable absorbers

Lukas Seitner,[1,*] Johannes Popp,[1] Michael Haider,[1]
Sukhdeep S. Dhillon,[2] Miriam S. Vitiello,[3] and Christian Jirauschek[1,4,†]

[1] *TUM School of Computation, Information and Technology, Technical University of Munich (TUM), D-85748 Garching, Germany*
[2] *Laboratoire de Physique de l'Ecole Normale Supérieure, ENS, Université PSL, CNRS, Sorbonne Université, Université de Paris, Paris, France*
[3] *NEST, CNR - Istituto Nanoscienze and Scuola Normale Superiore, Piazza San Silvestro 12, 56127, Pisa, Italy*
[4] *TUM Center for Quantum Engineering (ZQE), Technical University of Munich (TUM), D-85748 Garching, Germany*

I. MODEL DERIVATION

In the main paper, the reduced equations describing the gain are stated to be derived from the Bloch equations [1]. This section gives an overview of the derivation.

A. Bloch equations

Starting point are the Bloch equations of a two-level system, considering coherence $\eta_{21}^{\pm}$, inversion w, and inversion grating w^{+}:

$$\partial_t \eta_{21}^{\pm} = \mathrm{i}\Delta\eta_{21}^{\pm} - \frac{\mathrm{i}}{2\hbar} w d_{21} E^{\pm} - \frac{\mathrm{i}}{2\hbar} w^{\pm} d_{21} E^{\mp} - \gamma_2 \eta_{21}^{\pm}\,, \tag{1}$$

$$\partial_t w = \frac{2}{\hbar}\mathfrak{Im}\left\{ d_{21}\left[\eta_{21}^{+}\left(E^{+}\right)^{*} + \eta_{21}^{-}\left(E^{-}\right)^{*}\right]\right\} - \gamma_1 (w - w_{\mathrm{eq}})\,, \tag{2}$$

$$\partial_t w^{+} = \frac{\mathrm{i}}{\hbar}\left[d_{21}\left(\eta_{21}^{-}\right)^{*} E^{+} - d_{21}\eta_{21}^{+}\left(E^{-}\right)^{*}\right] - \gamma_1' w^{+}\,, \tag{3}$$

with $w^{-} = (w^{+})^{*}$. The remaining parameter, not yet mentioned in the main paper, is the detuning between the center frequency of the model and the resonance frequency of the two-level system $\Delta = \omega_{\mathrm{c}} - \omega_{21}$. In the here discussed case, $\Delta = 0$; however, it is taken into account for the sake of completeness.

B. Adiabatic Elimination

In a QCL, quantum mechanical dephasing of the coherent laser levels typically is the fastest process in the system. In the present model, this is captured by the fact that γ_2 is about one order of magnitude larger than γ_1. By assuming the dephasing to be instantaneous, we can perform an adiabatic elimination [2], which is formally obtained by integrating Eq. (1):

$$\begin{aligned} \eta_{21}^{\pm}(t) &= \eta_{21}^{\pm}(0)\exp[(\mathrm{i}\Delta - \gamma_2)t] \\ &\quad - \frac{\mathrm{i}}{2\hbar} d_{21}\int_0^t \exp[(\mathrm{i}\Delta-\gamma_2)\tau] w(t-\tau) E^{\pm}(t-\tau)\,\mathrm{d}\tau \\ &\quad - \frac{\mathrm{i}}{2\hbar} d_{21}\int_0^t \exp[(\mathrm{i}\Delta-\gamma_2)\tau] w^{\pm}(t-\tau) E^{\mp}(t-\tau)\,\mathrm{d}\tau\,. \end{aligned} \tag{4}$$

* lukas.seitner@tum.de; https://www.ee.cit.tum.de/cph
† jirauschek@tum.de; https://www.ee.cit.tum.de/cph

Considering that $\Delta = 0$, and assuming that w and $E^{\pm}$ vary slowly on a timescale of $1/\gamma_2$, we obtain for times $t \gg 1/\gamma_2$

$$\eta_{21}^{\pm}(t) = -\frac{\mathrm{i}}{2\hbar\gamma_2} d_{21}\left[w(t)E^{\pm}(t) + w^{\pm}(t)E^{\mp}(t)\right]. \tag{5}$$

According to the main text, $\eta_{21}^{\pm}$ is directly proportional to the polarization used in the propagation equation of the electric field.

In the following, the equations of motion for the inversion components w and w^+ are derived, with $w^- = (w^+)^*$. By plugging Eq. (5) into Eqs. (2) and (3), we eliminate the coherence term and remain with a set of two coupled differential equations. With the application of some calculation rules, these equations read

$$\partial_t w = -\frac{d_{21}^2}{\hbar^2\gamma_2}\left[w\big(|E^+|^2 + |E^-|^2\big) + \mathfrak{Re}\big\{w^+E^-(E^+)^* + w^-E^+(E^-)^*\big\}\right] - \gamma_1(w - w_{\mathrm{eq}}), \tag{6}$$

$$\partial_t w^+ = -\frac{d_{21}^2}{2\hbar^2\gamma_2}\left[w^+\big(|E^+|^2 + |E^-|^2\big) + 2wE^+(E^-)^*\right] - \gamma_1' w^+. \tag{7}$$

It is straightforward to see that each inversion contribution consists of a term proportional to the intensity $I \propto \big(|E^+|^2 + |E^-|^2\big)$, an interference term containing $E^+(E^-)^*$ and/or $E^-(E^+)^*$ and finally a recovery term containing the gain recovery rate γ_1.

Aiming for the simplest model that allows for stable pulses with $\tau_a \approx \tau_g$, we find by numerical evaluation, that in equations (6) and (7) terms containing a product of w^+ with intensity or interference have minor influence on the dynamics and can be disregarded since for reasonable intensities $|w^+| \ll w$ holds. The simplest set of equations holding sufficient information about the quantum system then results in

$$\partial_t w = -\frac{d_{21}^2}{\hbar^2\gamma_2} w\big(|E^+|^2 + |E^-|^2\big) - \gamma_1(w - w_{\mathrm{eq}}), \tag{8}$$

$$\partial_t w^+ = -\frac{d_{21}^2}{\hbar^2\gamma_2} wE^+(E^-)^* - \gamma_1' w^+, \tag{9}$$

as presented in the main paper.

C. Analytical considerations of directive gain

In the main paper, we discuss a directive gain of the QCL active region, which results from our simulation approach [see Eq. (10) of the main paper]. We further present analytical results of saturation intensities and gain components for two distinct scenarios [$I^- = I^+$; main paper Eq. (14), $I^{\mp} \ll I^{\pm}$; main paper Eq. (15)]. Here, we give a more detailed analytical derivation. In order to create a rather simple and intuitive scenario, we assume right- and left-traveling electric fields to have time-constant amplitudes $E^{\pm}(x)$. Setting $\partial_t = 0$ in Eqs. (6) and (7) and calculating in terms of Rabi-frequencies $\Omega^{\pm} = d_{21}E^{\pm}/\hbar$ yields

$$w^{\pm} = -\frac{2\Omega^{\pm}(\Omega^{\mp})^*}{2\gamma_1'\gamma_2 + (|\Omega^+|^2 + |\Omega^-|^2)} w, \tag{10}$$

$$w = \frac{\gamma_1\gamma_2}{\gamma_1\gamma_2 + |\Omega^+|^2 + |\Omega^-|^2 - \frac{4|\Omega^+|^2|\Omega^-|^2}{2\gamma_1'\gamma_2 + |\Omega^+|^2 + |\Omega^-|^2}} w_{\mathrm{eq}}. \tag{11}$$

Inserting Eq. (8) of the main text into Eq. (1) of the main text, we obtain

$$v_{\mathrm{g}}^{-1}\partial_t E^{\pm} \pm \partial_x E^{\pm} = \mathrm{i}\frac{n_{3\mathrm{D}}\omega_{\mathrm{c}}}{c_0\epsilon_0 n_0}\Gamma d_{21}\eta_{21}^{\pm} - lE^{\pm} + \left(D_f - \mathrm{i}\frac{\beta_2}{2}\right)\partial_t^2 E^{\pm}. \tag{12}$$

Expressing the polarization term by means of a gain coefficient, we obtain

$$\frac{1}{2}g^{\pm}E^{\pm} = \mathrm{i}\frac{n_{3\mathrm{D}}\omega_{\mathrm{c}}}{c_0\epsilon_0 n_0}\Gamma d_{21}\eta_{21}^{\pm} \qquad \Rightarrow \qquad g^{\pm} = 2\mathrm{i}\frac{\Gamma d_{21}^2 n_{3\mathrm{D}}\omega_{\mathrm{c}}}{c_0\epsilon_0 n_0\hbar\Omega^{\pm}}\eta_{21}^{\pm}. \tag{13}$$

Inserting Eqs. (5), (10), and (11), we obtain the power gain

$$g^{\pm} = \frac{\Gamma d_{21}^2 n_{3\mathrm{D}}\omega_{\mathrm{c}}}{\hbar\gamma_2 c_0\epsilon_0 n_0}\left(1 - \frac{2|\Omega^{\mp}|^2}{2\gamma_1'\gamma_2 + |\Omega^+|^2 + |\Omega^-|^2}\right) w. \tag{14}$$

Thus, the gain of the stronger field component is higher than for the weaker counterpropagating field. Obviously, in Eq. (14), only squared absolute values of the Rabi frequencies appear. Thus, we can make the transition to intensity as follows:

$$I^{\pm} = \frac{1}{2}\varepsilon_0 c_0 n_0 |E^{\pm}|^2 \,, \tag{15}$$

$$I_{\mathrm{s,g}} = \frac{\gamma_1\gamma_2\hbar^2\epsilon_0 n_0 c_0}{2d_{21}^2} \,, \tag{16}$$

$$\frac{I^{\pm}}{I_{\mathrm{s,g}}} = \frac{d_{21}^2|E^{\pm}|^2}{\gamma_1\gamma_2\hbar^2} \,, \tag{17}$$

$$|\Omega^{\pm}|^2 = \frac{I^{\pm}}{I_{\mathrm{s,g}}}\gamma_1\gamma_2 \,. \tag{18}$$

Inserting Eq. (18) into Eq. (14) yields Eq. (13) from the main paper.

As the prefactor in Eq. (14) is the same for both directions, but we are interested in the difference between the directions, we can arbitrarily set the prefactor to unity and obtain a normalized gain $g_{\mathrm{norm}}^{\pm}$, which can, with the use of Eq. (11), be rewritten as follows:

$$\begin{aligned} g_{\mathrm{norm}}^{\pm} &= \left(1 - \frac{2|\Omega^{\mp}|^2}{2\gamma_1'\gamma_2 + |\Omega^+|^2 + |\Omega^-|^2}\right) w \\ &= \left(\frac{2\gamma_1'\gamma_2 \pm |\Omega^+|^2 \mp |\Omega^-|^2}{2\gamma_1'\gamma_2 + |\Omega^+|^2 + |\Omega^-|^2}\right) \frac{\gamma_1\gamma_2}{\gamma_1\gamma_2 + |\Omega^+|^2 + |\Omega^-|^2 - \frac{4|\Omega^+|^2|\Omega^-|^2}{2\gamma_1'\gamma_2 + |\Omega^+|^2 + |\Omega^-|^2}} w_{\mathrm{eq}} \\ &= \frac{\gamma_1\gamma_2\left[2\gamma_1'\gamma_2 \pm \left(|\Omega^+|^2 - |\Omega^-|^2\right)\right]}{(\gamma_1\gamma_2 + |\Omega^+|^2 + |\Omega^-|^2)(2\gamma_1'\gamma_2 + |\Omega^+|^2 + |\Omega^-|^2) - 4|\Omega^+|^2|\Omega^-|^2} w_{\mathrm{eq}} \\ &= \frac{\gamma_1\gamma_2\left[2\gamma_1'\gamma_2 \pm \left(|\Omega^+|^2 - |\Omega^-|^2\right)\right]}{2\gamma_1'\gamma_1\gamma_2^2 + (2\gamma_1'\gamma_2 + \gamma_1\gamma_2)(|\Omega^+|^2 + |\Omega^-|^2) + \left(|\Omega^+|^2 - |\Omega^-|^2\right)^2} w_{\mathrm{eq}} \,. \end{aligned} \tag{19}$$

Now, we consider a pulse with a long duration compared to the gain recovery dynamics, such that the above solutions are still approximately valid. First, we investigate the case with the pulse being located close to a facet. Assuming full reflectance, we have $|\Omega^-| = |\Omega^+|$ in the region close to the facet. Inserting this condition into Eq. (19), we obtain

$$g_{\mathrm{norm}}^{\pm} = \frac{w_{\mathrm{eq}}}{1 + \frac{2|\Omega^+|^2}{\left(\frac{2\gamma_1\gamma_1'\gamma_2}{2\gamma_1'+\gamma_1}\right)}} = \frac{w_{\mathrm{eq}}}{1 + \frac{2I^+}{\left(\frac{2\gamma_1'}{2\gamma_1'+\gamma_1}\right)I_{\mathrm{s,g}}}} \,. \tag{20}$$

Here, we also made the transition from Rabi frequencies to intensities by using Eq. (18). By comparison of coefficients, the saturation intensity deviates from its original definition by the factor $2\gamma_1'/(2\gamma_1' + \gamma_1)$, as presented in Eq. (14) of the main paper. This yields a reduction of the saturation intensity to 2/3 of its original value in presence of full SHB ($\gamma_1' = \gamma_1$) and equally strong fields $I^+ = I^-$.

Now, let us consider the case with highly asymmetric field amplitudes of the counterpropagating components. Such a scenario appears when the pulse peak is located in the middle of the cavity. Inserting the condition $|\Omega^-| \ll |\Omega^+|$ into Eq. (19) yields

$$\begin{aligned} g_{\mathrm{norm}}^{\pm} &= \frac{\gamma_1\gamma_2\left(2\gamma_1'\gamma_2 \pm |\Omega^+|^2\right)}{2\gamma_1'\gamma_1\gamma_2^2 + (2\gamma_1'\gamma_2 + \gamma_1\gamma_2)|\Omega^+|^2 + |\Omega^+|^4} w_{\mathrm{eq}} \\ &= \frac{1}{1 + \frac{|\Omega^+|^2}{\gamma_1\gamma_2}} w_{\mathrm{eq}} \frac{2\gamma_1'\gamma_2 \pm |\Omega^+|^2}{2\gamma_1'\gamma_2 + |\Omega^+|^2} = \frac{1}{1 + \frac{I^+}{I_{\mathrm{s,g}}}} w_{\mathrm{eq}} \frac{2\gamma_1'/\gamma_1 \pm I^+/I_{\mathrm{s,g}}}{2\gamma_1'/\gamma_1 + I^+/I_{\mathrm{s,g}}} \,. \end{aligned} \tag{21}$$

Here, the saturation intensity remains at its initial value, as without SHB since basically no interference effects arise. But Ω^- sees a gain reduction by a factor

$$g_{\mathrm{norm}}^{-} = g_{\mathrm{norm}}^{+} \frac{2\gamma_1'\gamma_2 - |\Omega^+|^2}{2\gamma_1'\gamma_2 + |\Omega^+|^2} = g_{\mathrm{norm}}^{+}\left(1 - \frac{2I^+/I_{\mathrm{s,g}}}{2\gamma_1'/\gamma_1 + I^+/I_{\mathrm{s,g}}}\right) , \tag{22}$$

corresponding to Eq. (15) of the main paper. For strong $|\Omega^+|^2$, this can even become a loss.

D. Absence of population grating

As stated in the main paper, if the absorber recovery is significantly faster than the gain recovery, $\tau_\mathrm{a} < \tau_\mathrm{g}$, then pulse formation is explainable without the effects of optical interference. If the inversion grating w^+ is disregarded, the overall inversion is independent of interference terms and exclusively governed by the intensity-dependent term of Eqs. (8) and (9), yielding

$$w^+ = 0\,, \tag{23}$$

$$\partial_t w = -\frac{d_{21}^2}{\hbar^2\gamma_2} w\big(|E^+|^2 + |E^-|^2\big) - \gamma_1(w - w_\mathrm{eq})\,, \tag{24}$$

$$\eta_{21}^\pm(t) = -\frac{\mathrm{i}d_{21}}{2\hbar\gamma_2} w(t)E^\pm(t)\,. \tag{25}$$

In this case, the equations can be re-written in the form of the absorber equations, such that the gain results as an incoherent saturable absorber with negative loss, here described by the coefficient $a_\mathrm{g} > 0$, according to

$$f^\pm = \frac{a_\mathrm{g}}{2}E^\pm\,, \tag{26}$$

$$\partial_t a_\mathrm{g} = -\gamma_1\frac{I}{I_\mathrm{s,g}}a_\mathrm{g} - \gamma_1(a_\mathrm{g} - a_\mathrm{g,0})\,. \tag{27}$$

The following equalities are used in this case

$$a_\mathrm{g} = \Gamma\frac{n_\mathrm{3D}\omega_\mathrm{c}}{\epsilon_0 n_0 c_0\hbar\gamma_2} d_{21}^2 w\,, \tag{28}$$

$$a_\mathrm{g,0} = \Gamma\frac{n_\mathrm{3D}\omega_\mathrm{c}}{\epsilon_0 n_0 c_0\hbar\gamma_2} d_{21}^2 w_\mathrm{eq}\,, \tag{29}$$

$$I(z,t) = \frac{\epsilon_0 c_0 n_0}{2}\left[|E^+(x,t)|^2 + |E^-(x,t)|^2\right], \tag{30}$$

$$I_\mathrm{s,g} = \frac{\gamma_1\gamma_2\hbar^2\epsilon_0 n_0 c_0}{2d_{21}^2}\,. \tag{31}$$

II. MICROSCOPIC PARAMETERS

This section intends to give an overview of the microscopic parameters used for the two-level system, in Tab. I.

Table I. Parameters describing the two-level quantum system consisting of upper (ULL) and lower laser level (LLL).

Parameter	Abbreviation	Value
Resonance frequency	ω_{21}	$2\pi \cdot 3\,\mathrm{THz}$
Dipole moment	d_{21}	$6.02\,\mathrm{nm} \times e$
Scattering rate ULL → LLL	r_{21}	$1.5 \times 10^{11}\,\mathrm{s}^{-1}$
Scattering rate LLL → ULL	r_{12}	$2.3 \times 10^{11}\,\mathrm{s}^{-1}$
Dephasing rate	γ_2	$6 \times 10^{12}\,\mathrm{s}^{-1}$

From the scattering rates, the gain recovery rate γ_1 and the equilibrium inversion can be calculated according to

$$\gamma_1 = r_{21} + r_{12}\,, \tag{32}$$

$$w_\mathrm{eq} = \frac{r_{12} - r_{21}}{\gamma_1}\,. \tag{33}$$

To complete the description of the system, the remaining parameters of the used cavity are stated in Tab. II. The value of background group velocity dispersion was increased slightly to $7.5 \times 10^{-23}\,\mathrm{s}^2\,\mathrm{m}^{-1}$ in the simulation for Fig. 2 of the main paper, as it yielded the best match for the temporal pulse width.

Table II. Parameters describing the cavity.

Parameter	Abbreviation	Value
Length		2.37 mm
Mirror reflectivity		1
Overlap Factor	Γ	0.97
Doping density	$n_{3\mathrm{D}}$	$3.8 \times 10^{21}\,\mathrm{m}^{-3}$
Background group velocity dispersion	β_2	$5 \times 10^{-23}\,\mathrm{s}^2\,\mathrm{m}^{-1}$
Background refractive index	n_0	3.6

III. ADDITIONAL RESULTS

A. Electric field without graphene saturable absorber

As a reference to the pulsed operation of the QCL presented in the main paper, this section presents the results without graphene saturable absorber. In the simulation, this scenario is realized by setting the saturable loss to $a_1 = 0$. It is clearly visible in Fig. 1 that the experiment and simulation yield chaotic outputs without background suppression.

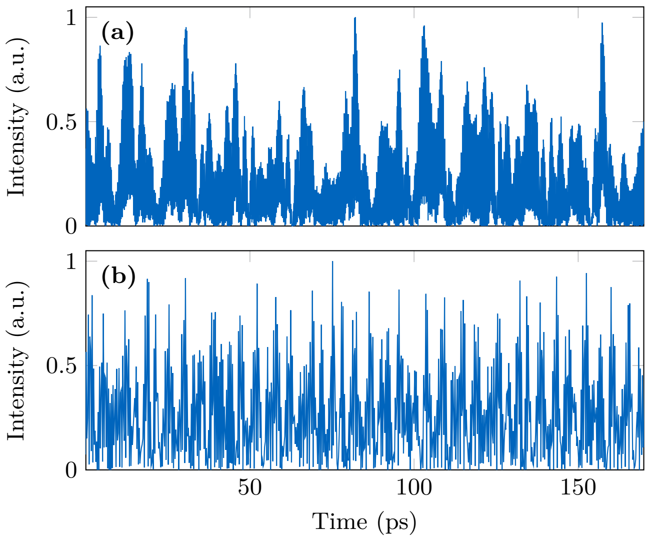

Figure 1. Comparison of **(a)** simulation results to **(b)** experiment in absence of a saturable absorber realized by graphene.

B. Influence of waveguide dispersion

As mentioned in the discussion of Fig. 5 of the main paper, chromatic dispersion has a significant influence on the width and peak intensity of a pulse. Figure 2 shows simulation results with different group velocity dispersion parameters β_2.

C. Simulation with long gain recovery

This section intends to give an overview of the parameters used for the simulations with gain recovery time $\tau_\mathrm{g} = 10.5\,\mathrm{ps}$. The values used for the gain medium are listed in Tab. III, and for the absorber in Tab. IV. According to Eq. (31) the saturation intensity of the gain reduces when the recovery time gets increased. Therefore, the absorber saturation intensity must be adapted. Furthermore, we change the dephasing rate γ_2 in order to show the stability of the model against this parameter. In order to keep the peak gain constant, the dipole moment is reduced to $4.26\,\mathrm{nm} \times e$.

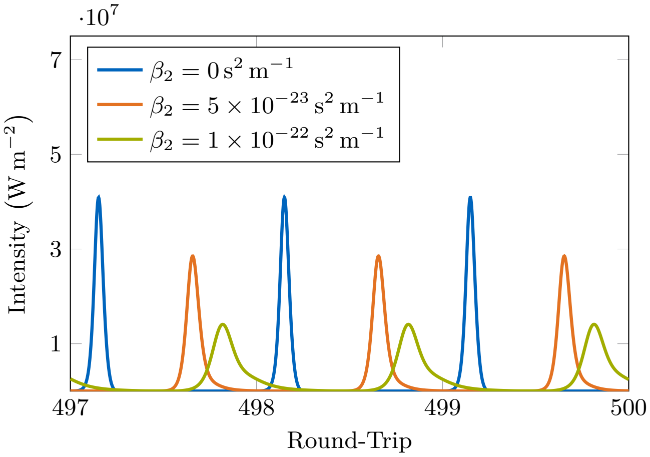

Figure 2. Comparison of simulation results with different chromatic dispersion.

Table III. Alternative parameters of gain

$1/\gamma_1$	$1/\gamma_2$	g_0	$I_{\mathrm{s,g}}$
10.53 ps	0.333 ps	$22.5\,\mathrm{cm^{-1}}$	$3.26 \times 10^7\,\mathrm{W\,m^{-2}}$

Table IV. Alternative parameters of absorber

τ_{a}	a_0	a_1	$I_{\mathrm{s,a}}$
3 ps	$11.0\,\mathrm{cm^{-1}}$	$10.5\,\mathrm{cm^{-1}}$	$2.5 \times 10^7\,\mathrm{W\,m^{-2}}$

The resulting formation and propagation behavior of the pulse is shown in Fig. 3. Compared to the case of similar recovery times of absorber and gain that is discussed in the main paper, here, the pulse starts up faster, and the net gain has stronger deviations from zero.

D. Pulse stability at varying recovery

In the main text it was briefly mentioned, that for longer gain recovery times, pulses are stable at higher pumping levels. In Fig. 4 the maximum ratio between gain and loss that still yields stable pulses is plotted, for the presence and absence of w^+ (spatial hole burning). It is visible, that with increasing gain recovery time and constant absorber recovery time, the range for stable pulses significantly increases. When τ_{g} approaches the round-trip time the model does not require any effects beyond the standard passive mode-locking description. However, when both recovery times are similar, the spatial hole burning becomes crucial, and only a small range of unsaturated gain values above the lasing threshold yields single pulses before breakup to multi-pulsing. We note that the cavity length was reduced to 2 mm in order to increase the numerical speed for the evaluation.

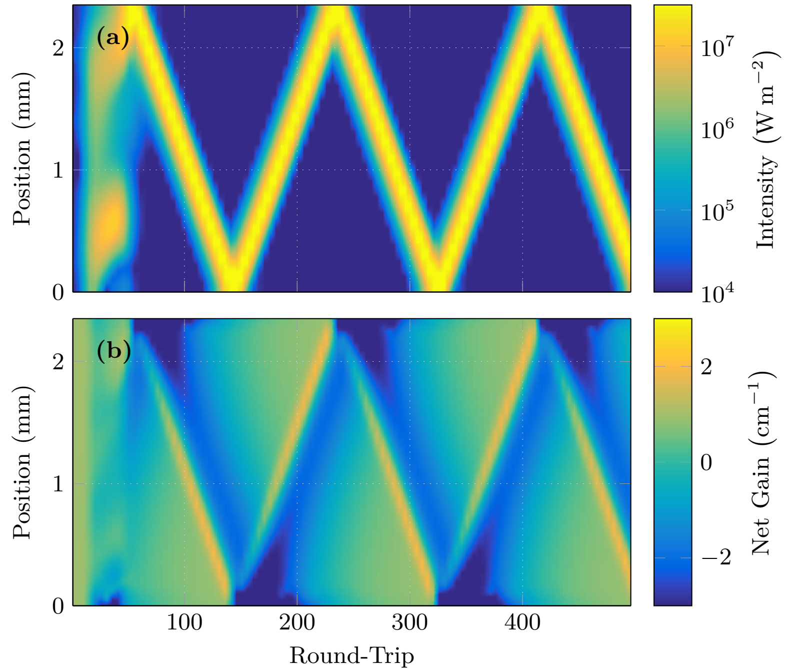

Figure 3. Visualization of **(a)** intra-cavity intensity and **(b)** net gain during the formation process for a gain recovery time $\tau_g = 10.5\,\mathrm{ps}$.

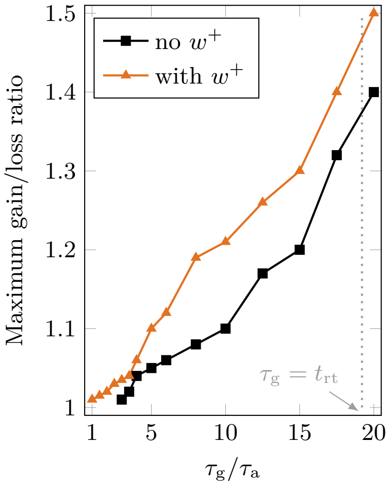

Figure 4. Maximum ratio of gain and loss that still yields stable pulses for different gain recovery times.

[1] C. Y. Wang, L. Diehl, A. Gordon, C. Jirauschek, F. X. Kärtner, A. Belyanin, D. Bour, S. Corzine, G. Höfler, M. Troccoli, J. Faist, and F. Capasso, Phys. Rev. A **75**, 031802(R) (2007).
[2] L. Lugiato, F. Prati, and M. Brambilla, *Nonlinear Optical Systems* (Cambridge University Press, 2015).
